# Comparing the Effect of Incentive Spirometry with Acapella on Blood Gases in Physiotherapy After Coronary Artery Bypass Graft

**DOI:** 10.7759/cureus.6851

**Published:** 2020-02-03

**Authors:** Masood Alam, Shafqat Hussain, Muhammad Imran Shehzad, Azam Mushtaq, Abdul Rauf, Sohaib Ishaq

**Affiliations:** 1 Pulmonology, Chaudhary Pervaiz Elahi Institute of Cardiology, Multan, PAK; 2 Cardiac Surgery, Chaudhary Pervaiz Elahi Institute of Cardiology, Multan, PAK; 3 Pulmonology, Quaid-e-Azam Medical College, Bahawalpur, PAK; 4 Pulmonology, Dera Ghazi Khan Teaching Hospital, Dera Ghazi Khan, PAK; 5 Internal Medicine: Critical Care, Services Hospital, Lahore, PAK

**Keywords:** incentive spirometry, acapella, physiotherapy, coronary artery bypass graft

## Abstract

Objective

To compare the effect of incentive spirometry with Acapella (Smiths Medical Inc, Carlsbad, California) in physiotherapy after coronary artery bypass surgery.

Methods

A randomized controlled trial comparing incentive spirometry with Acapella was conducted in the intensive care unit of Chaudhary Pervaiz Elahi Institute of Cardiology (CPEIC) Multan. The study began from December 2017 to August 2019 after getting approval from the ethical committee of the hospital. Informed written consent was taken from all 270 patients who were included in the study. Patients who underwent coronary artery bypass graft (CABG) were divided into two groups by the lottery method. The primary end-point of the study was to check the blood gases on Day 3 after the procedure at room air and compare it with the baseline and with blood gases immediately after the procedure. SPSS 23 (IBM Corp., Armonk, NY) was used to analyze the data of this study. For qualitative variables in data such as gender, place of living, patients with any comorbidities, and education status were statistically analyzed in percentage and frequencies. For numerical variables, such as age, body mass index, blood gases values, distance covered in a six-minute walk test, and spirometry values were analyzed and statistically measured as mean and standard deviation. A P-value of less than .05 was considered significant.

Results

The mean partial pressure of oxygen (PaO2) of incentive spirometry was 58.1±2.31 and 67.2±3.24 after extubation and after three days, respectively. While the PaO2 of Acapella was 56.3±3.43 and 66.4±3.54 after extubation and after three days, respectively. The mean PCO2 of incentive spirometry was 41.4±3.26 and 36.1±2.11 after extubation and after three days, respectively. While the partial pressure of carbon dioxide (PCO2) of Acapella was 39.4±2.55 and 37.5±3.58 after extubation and after three days, respectively. The differences were statistically significant at p-value ≤0.05.

Conclusion

It was concluded that both Acapella and incentive spirometry treatment after coronary artery bypass graft improved blood gases.

## Introduction

The removal of respiratory secretions is an important concern during the rehabilitation phase after cardiac surgery and postoperative pain can interfere with the elimination of respiratory tract secretions. The secretions of the respiratory tract cannot be eliminated, producing atelectasis, pneumonia, and increased mortality [1]. Hand stroke with chest wall vibration is a widely accepted method of physiotherapy, which helps cleanse the respiratory system. However, this requires a lot of manpower, depends on the operator, and consumes a lot of time. Moreover, it may be painful for patients immediately after the operation. Respiratory problems are still the main cause of morbidity in postoperative heart surgery and long-term hospitalization [2]. However, patients with mitral valve disease have a higher risk of respiratory complications after cardiac surgery than patients with coronary artery disease [3]. Postoperative pulmonary complications (PPCs) were defined as "Any pulmonary abnormality that occurs in the postoperative period and produces identifiable diseases or dysfunctions that are clinically significant and negatively affect the clinical course" [4]. The increase in the hospitalization period is also an indirect measure of postoperative pulmonary complications [5].

There are several reports that alternative types of vibration and oscillation can facilitate air removal and improve lung function in various clinical settings [6-10]. So far, no clinical studies have evaluated the effect of positive vasodilator pressure with Acapella (Smiths Medical Inc, Carlsbad, California) in patients undergoing coronary artery bypass grafting. Acapella consists of an anti-weight cover and a metal bar connected to a handle and magnet. The oscillation of the airflow is generated by breaking and repairing the magnetic gravity through the plug where the air passing through the unit is interrupted periodically. Patients in the control cluster were treated with incentive spirometry, which is routinely utilized after a cardiac procedure in our hospital. Incentive spirometry is intended to encourage the patient to take a full breath, impersonating unconstrained yawning or moaning, expanding or keeping up breathed-in lung volume, and enhancing sputum expectoration [11]. Patients were told to take 10 profound inward breaths with their lips fixed around the mouthpiece - this was propelled by the visual motivation furnished by the balls ascending with the dynamic inward breath. They were urged to utilize incentive spirometry every two hours, in a mediocre sitting position while conscious.

The purpose of this study is to investigate and compare the two methods of physiotherapy and rehabilitation and their effects on pulmonary function and blood gases. This research will provide a base for further investigation and will also establish some facts.

## Materials and methods

A randomized controlled trial comparing incentive spirometry with Acapella was conducted in the intensive care unit of CPEIC Multan. The study began after getting approval from the ethical committee of the hospital and the period of the study was from December 2017 to August 2019. Informed written consent was taken from all 270 patients who were included in the study. Patients who underwent coronary artery bypass grafting (CABG) were divided into two groups by the lottery method. The primary end-point of the study was to check the blood gases on Day 3 after the procedure at room air and compare it with the baseline and with blood gases immediately after the procedure. The secondary end-point of the study was to check the functional status of patients in both groups on Day 3 after surgery by a six-minute walk test. Group 1 will include 135 of those patients who received standard physiotherapy, that is, breathing exercise, huffing, coughing, and manual percussion along with incentive spirometry and other 135 Group 2 patients received the same standard physiotherapy and Acapella instead of incentive spirometry. The exclusion criteria of this study was patients with a body mass index (BMI) of less than 16kg/m^2^ and greater than 35kg/m^2^; patients who had a baseline partial pressure of oxygen (PaO2) less than 75 mm of Hg and partial pressure of carbon dioxide (PCO2) greater than 45mm of Hg; patients who required more than 48 hours of intubation after surgery; patients who needed re-intubation; patients who had history of respiratory tract infection within a period of three months and patients with any neuromuscular disease. The study done by Cho et al. was taken as a reference study to calculate the sample size for which a 95% confidence interval and a study strength of 80 will be taken [12].

All patients of age more than 30 years old admitted to the hospital for CABG were registered for the study. Their history was taken to fully evaluate them, a clinical examination was carried out to assess any comorbidities, and the vitals of patients were recorded. One day before surgery, an arterial blood sample of all patients was obtained from the radial artery to document the baseline blood gas situation and, on the same day, spirometry of all the patients was carried out and their baseline forced vital capacity (FVC) and forced expiratory volume (FEV1) were documented. The patient’s functional status was checked at the time of admission by the six-minute walk test. After the procedure and extubation, an arterial blood gas analysis was performed. And on Day 3, arterial blood gas analysis was performed again and functional status was checked by the six-minute walk test.

SPSS 23 (IBM Corp., Armonk, NY) was used to analyze the data of this study. For qualitative variables in data such as gender, place of living, patients with any comorbidities, and education status were statistically analyzed in percentage and frequencies. For numerical variables, such as age, BMI, blood gases values, distance covered in the six-minute walk test, and spirometry values were analyzed and statistically measured as mean and standard deviation. The chi-square and t-test were applied to check the significance. And a p-value of equal or less than .05 was considered significant.

## Results

Two hundred and seventy patients were included in this study. The patients were randomized into two groups: incentive spirometry and Acapella. The mean age and BMI of the incentive spirometry were 44.63±3.38 years and 26.37±2.31 kg/m^2^, respectively; n=54 (40%) lived in rural areas' and n=81 (60%) lived in urban areas. There were n=47 (34.8%) smokers. Diabetes mellitus (DM) and hypertension (HTN) were observed as n=63 (46.7%) and n=52 (38.5%), respectively. While the mean age and BMI of the Acapella were 49.14±3.27 years and 25.48±1.74 kg/m^2^, respectively. N=51 (37.8%) lived in rural areas and n=84 (62.2%) lived in urban areas. There were n=64 (47.4%) smokers. DM and HTN were observed as n=58 (43%) and n=46 (34.1%), respectively. The difference was statistically significant for smoking status (p=0.035) (Table 1).

**Table 1:** Demographic characteristics among the groups BMI: body mass index; DM: diabetes mellitus; HTN: hypertension

Variable	Incentive Spirometry n=135	Acapella n=135	P-value
Age (years)	44.63±3.38	49.14±3.27	0.712
BMI (kg/m^2^)	26.37±2.31	25.48±1.74	0.000
DM	n=63 (46.7%)	n=58 (43%)	0.541
HTN	n=52 (38.5%)	n=46 (34.1%)	0.448
Smokers	n=47 (34.8%)	n=64 (47.4%)	0.035
Residential status			
Rural	n=54 (40%)	n=51 (37.8%)	0.708
Urban	n=81 (60%)	n=84 (62.2%)	

The mean PaO2 of incentive spirometry and Acapella was 70.95±2.13 mmHg and 68.62±2.81 mmHg, respectively, before surgery. The difference was statistically significant (p=0.000). The mean PCO2 of incentive spirometry and Acapella was 38.17±1.72 mmHg and 37.73±2.59 mmHg, respectively. The difference was statistically insignificant (p=0.099). The mean six-minute-walk test of incentive spirometry and Acapella was 280.47±6.12meters and 295.89±4.83meters, respectively. The difference was statistically significant (p=0.000). FVC (%) was found to be n=92 (68.1%) and n=94 (69.6%) for the incentive spirometry and Acapella groups, respectively. FEV1 was found to be n=92 (68.1%) and n=80 (59.3%) for the incentive spirometry and Acapella groups, respectively. But, these differences were not statistically significant: (p=0.793) and (p=0.129), respectively (Table 2).

**Table 2:** Parameters before surgery forced vital capacity (FVC); forced expiratory volume (FEV1); partial pressure of oxygen (PaO2); partial pressure of carbon dioxide (PCO2)

Variable	Incentive Spirometry n=135	Acapella n=135	P-value
FVC (%)	n=92 (68.1%)	n=94 (69.6%)	0.793
FEV_1 (%)_	n=92 (68.1%)	n=80 (59.3%)	0.129
PaO_2 (mmHg)_	70.95±2.13	68.62±2.81	0.000
PcO_2 (mmHg)_	38.17±1.72	37.73±2.59	0.099
Six-minute walk test (meters)	280.47±6.12	295.89±4.83	0.000

The mean PaO2 of incentive spirometry was 58.1±2.31 mmHg and 67.2±3.24 mmHg after extubation and after three days, respectively. While the PaO2 of Acapella was 56.3±3.43 mmHg and 66.4±3.54 mmHg after extubation and after three days, respectively. The mean PCO2 of incentive spirometry was 41.4±3.26 mmHg and 36.1±2.11 mmHg, after extubation and after three days, respectively. While, the PCO2 of Acapella was 39.4±2.55 mmHg and 37.5±3.58 mmHg, after extubation and after three days, respectively. The differences were statistically significant (Table 3). P-value ≤0.05 was considered significant.

**Table 3:** Parameters after surgery partial pressure of oxygen (PaO2); partial pressure of carbon dioxide (PCO2)

Variable	After extubation	After 3 days	P-value
PaO_2 _(mmHg)			
Incentive spirometry	58.1±2.31	67.2±3.24	0.000
Acapella	56.3±3.43	66.4±3.54	0.000
PCO_2 _(mmHg)			
Incentive spirometry	41.4±3.26	36.1±2.11	0.000
Acapella	39.4±2.55	37.5±3.58	0.000

## Discussion

In this study, treatment with incentive spirometry and Acapella improved PaO2 on the third day after extubation. Similarly, both caused a significant change in PaCO2 in both groups.

In some studies, it was recommended to use physiotherapy to decrease the stay in the hospital and decrease complications, especially after thoracic surgery [13]. Different kinds of physiotherapy of the chest have been investigated and many findings have been published. High-frequency oscillation, combined with expiratory positive pressure, has caused increased clearance and sputum expectoration in bronchiectasis and in patients who were on mechanical ventilation [14-17]. In one study, it was published that in patients who underwent lobectomy, the high-frequency oscillation of the chest improved oxygenation and lung function after surgery [18]. The manufacturer of the Acapella device claimed this manoeuver. So, it was assumed that it would cause a similar clearance of airways and would improve lung function after lung surgery.

The use of Acapella was comfortable as compared to incentive spirometry. In this investigation, the effect of Acapella on drains was not studied. As in some previous research, it was noted that oscillation of the chest wall with high-frequency oscillation was associated with more drainage in the chest tube [18]. As Acapella transmits oscillations and vibrations internally, it did not cause harm to the wound and was safer as compared to manual percussion of the chest wall.

In the previous study, oscillation and vibration of the chest wall improved oxygenation and FEV1 in patients who underwent lobectomy as compared to standard physiotherapy [18]. In our study, incentive spirometry and Acapella both improved blood gases. As both groups also received standard physiotherapy in addition to these interventions, it could be due to this that both groups achieved significant improvement.

In this study, patients who required prolonged mechanical ventilation were excluded. The limitation of our study was that after surgery, lung function tests were not done to document the change in lung volumes and capacities. Most of them were lost to follow-up. In this study, on the third day, blood gases values were obtained and statistically analyzed and days between them were not included in the research. Time of anesthesia was also not considered in this investigation, which is also one of the main factors that increase the secretion and atelectasis of lung. Similarly, patients who had limited effort were treated with steam nebulization and more sessions of manual percussion were done on these patients. But this factor was ignored in this research.

## Conclusions

The results of our study concluded that both Acapella and incentive spirometry have the tendency to improve oxygenation after cardiac bypass surgery. There was no significant difference between both of these techniques in improving oxygenation. Any of the techniques can be used postoperatively to improve oxygenation.
